# Seat Occupancy Detection Based on a Low-Power Microcontroller and a Single FSR †

**DOI:** 10.3390/s19030699

**Published:** 2019-02-08

**Authors:** Ernesto Sifuentes, Rafael Gonzalez-Landaeta, Juan Cota-Ruiz, Ferran Reverter

**Affiliations:** 1 Department of Computer and Electrical Engineering, Universidad Autónoma de Ciudad Juárez (UACJ), Ciudad Juárez 32310, Mexico; esifuent@uacj.mx (E.S.); jcota@uacj.mx (J.C.-R.); 2 Department of Electronic Engineering, Universitat Politècnica de Catalunya (UPC)–BarcelonaTech, Castelldefels, 08860 Barcelona, Spain; ferran.reverter@upc.edu

**Keywords:** autonomous sensor, force sensing resistor, microcontroller, resistive sensor, seat occupancy detection, sensor interface electronics

## Abstract

This paper proposes a microcontroller-based measurement system to detect and confirm the presence of a subject in a chair. The system relies on a single Force Sensing Resistor (FSR), which is arranged in the seat of the chair, that undergoes a sudden resistance change when a subject/object is seated/placed over the chair. In order to distinguish between a subject and an inanimate object, the system also monitors small-signal variations of the FSR resistance caused by respiration. These resistance variations are then directly measured by a low-cost general-purpose microcontroller unit (MCU) without using either an analogue processing stage or an analogue-to-digital converter. Two versions of such a MCU-based circuit are presented: one to prove the concept of the measurement, and another with a smart wake-up (generated by the sudden resistance change) intended to reduce the energy consumption. The feasibility of the proposed measurement system is experimentally demonstrated with subjects of different weight sitting at different postures, and also with objects of different weight. The MCU-based circuit with a smart wake-up shows a standby current consumption of 800 nA, and requires an energy of 125 µJ to carry out the measurement after the wake-up.

## 1. Introduction

Seat-occupancy monitoring systems generally use mechanical sensors that detect weight, pressure, force or acceleration over the seat. These mechanical sensors can be resistive [1], capacitive [2] or inductive [3], but the former are the most common. Different types of resistive mechanical sensors can be employed, for instance: metallic strain gauges, semiconductor strain gauges and force sensing resistors (FSR), the latter being the cheapest and the most easily integrated into the chair structure. Mechanical sensors, however, have difficulties in distinguishing between subjects and objects seated/placed over the seat. This is usually solved by comparing the set of data to known patterns [4] or by combining the information of different types of sensor. For example, in [5], the information of the resistive mechanical sensor is combined with that provided by two additional sensors: a thermal sensor and a capacitive proximity sensor. The combination of proximity sensors, instead of mechanical sensors, have also been reported for seat-occupancy monitoring systems, as suggested in [6] by combining inductive and capacitive proximity sensors.

Resistive mechanical sensors, integrated in wheelchairs and conventional chairs, also play a significant role in health telemonitoring applications. However, several sensors are generally required to carry out the measurement of interest. For instance, the sitting posture has been proposed to be detected by employing: four load cells arranged in the seat of a chair in [7] and twelve FSRs distributed in both the seat and the backrest of a wheelchair in [8]. The respiratory signal has also been monitored using resistive mechanical sensors, for example: four FSRs attached to the backrest of a conventional chair in [9], and nine FSRs distributed in both the seat and the backrest of a wheelchair in [10].

This paper, which continues and expands the work presented in [11], proposes a novel measurement method for seat occupancy detection and classification using a single FSR. The additional information usually required for the subject-object classification is extracted from the FSR itself, which is exploited to monitor the respiratory signal similarly to the applications described in the previous paragraph. It is expected that the occupancy causes a large-signal variation of the FSR, whereas the respiration generates a small-signal variation enabling the confirmation of the presence of a subject rather than an object.

As for the read-out electronic circuit, it is proposed to read both large- and small-signal variations of the FSR applying the concept of direct interface circuit (DIC) [12], where the sensor is directly connected to a low-cost microcontroller unit (MCU) without using intermediate analogue electronics either an analogue-to-digital converter (ADC). These MCU-based circuits have been extensively analyzed and proved for resistive [13,14,15], capacitive [16,17,18] and inductive [19,20] sensors with different topologies, but not for resistive sensors undergoing both large- and small-signal variations, as we have in the application of interest here. DICs have also been suggested for sensors providing a quasi-static analogue output voltage [21,22] and as a versatile interface circuit for the measurement of different types of sensor [23].

According to the last two paragraphs, the main contribution of this paper is the proposal of a MCU-based circuit to detect and confirm the presence of a subject seated over a chair using a single FSR. The same sensor is employed to wake-up the MCU in the presence of a subject/object, and then to monitor the respiratory signal to confirm the presence of a subject. Moreover, this is carried out without using any intermediate analogue electronics, thus resulting in a low-power, low-cost design solution. Consequently, the proposed method is attractive for autonomous sensor applications that require the detection and confirmation of people sitting in chairs, such as intelligent airbag deployment systems and aircraft boarding systems.

The paper is organized as follows. Section 2 describes the operating principle of the seat occupancy detection and classification technique. Section 3 explains how the FSR is measured through the DIC and proposes an improved version with a smart wake-up so as to reduce its energy consumption. Section 4 describes the setup and the measurement method. Section 5 shows and discusses the experimental results. Finally, Section 6 draws the main conclusions.

## 2. Operating Principle

The proposed method for the seat occupancy detection and classification relies on a single FSR attached at the center of the seat. A FSR is a sensor whose resistance (*R_x_*) decreases with increasing the force applied to it; in the application of interest, the force will be exerted by the subject/object seated/placed over the chair. This is a very low-cost, thin-size resistive mechanical sensor that can be easily integrated into the chair structure; however, it has limitations in terms of accuracy, linearity, interchangeability and time drifts. The repeatability due to time drifts can be quite critical when the FSR is subjected to a static loading for a long time interval (say, some hours), but it is quite acceptable (at least for FSRs from Interlink Electronics) when the FSR is subjected to a dynamic loading for a short time interval (say, a few seconds) [24]. The previous limitations are not expected to be critical here since the aim is to monitor resistance variations due to respiration in a few seconds, and not to determine the exact value of the subject/object weight nor the respiratory rate.

**Figure 1:**
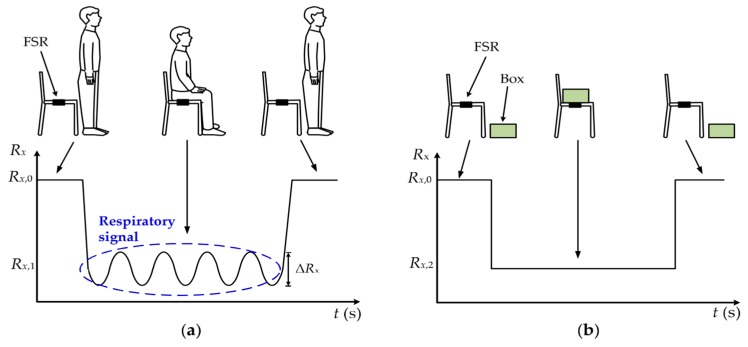
FSR variations when over the chair we have: (**a**) a subject; (**b**) an object.

The operating principle of the proposed measurement method is shown in Figure 1, where three scenarios are possible: (1) The seat is vacant: *R_x_* remains constant with respect to *R_x_*_,0_, which is the nominal resistance under zero-force conditions. (2) A subject is seated over the chair: *R_x_* suddenly decreases (e.g., from *R_x_*_,0_ to *R_x_*_,1_) due to the subject’s weight. Then, as the subject stays there, his/her respiration causes small-signal variations of *R_x_* (∆*R_x_* in Figure 1a) that enables us to confirm the presence of a subject. (3) An inanimate object is placed over the chair: *R_x_* undergoes a sudden decrease due to the object’s weight, but remains constant at *R_x_*_,2_, as shown in Figure 1b. Therefore, according to Figure 1, if the measurement system is able to detect such small-signal variations caused by respiration, the subject-object classification can be carried out using a single resistive mechanical sensor.

## 3. Read-out Interface Circuit

The FSR variations shown in Figure 1 are proposed to be measured by a low-cost general-purpose MCU applying the DIC concept [12]. The MCU only needs to have an embedded digital timer and a few input/output (I/O) digital port pins; no analogue (e.g., comparator) or mixed (e.g., ADC) embedded peripherals are required. Next, two proposals for the MCU-based circuit are presented. The first one is a basic topology that will enable us to prove the measurement method introduced in Section 2, whereas the second one is an energy-efficient topology with a smart wake-up.

### 3.1. Basic Read-out Circuit

**Figure 2:**
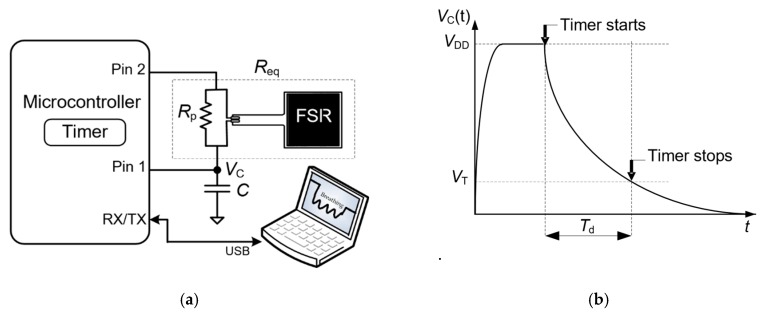
(**a**) DIC applied to measure the FSR so as to detect the occupancy of a seat; (**b**) waveform of the voltage across *C* during the measurement of the FSR.

The basic topology of the DIC to read the FSR is shown in Figure 2a. First of all, note that the FSR has a resistor (*R*_p_) in parallel so as to linearize its response and also to avoid long measurements in zero-force conditions. The operating principle of this circuit is as follows. Initially, Pin 1 provides a digital ‘1’ and Pin 2 is in high-impedance (HZ) state and, consequently, the well-known capacitor *C* is quickly charged to the supply voltage (*V*_DD_). Next, Pin 1 is in HZ and Pin 2 provides a digital ‘0’ so that *C* is discharged towards ground through the equivalent resistance (*R*_eq_ = *R*_p_‖*R_x_*). In the meantime, an embedded timer (which uses a high-frequency clock signal as a reference) measures the time interval required to do the discharge. When the exponential discharging voltage crosses the threshold voltage (*V*_T_) of the digital buffer embedded into Pin 1, the timer is stopped, as shown in Figure 2b. The resulting digital number stored in the timer is proportional to the discharging time (*T*_d_) and also to *R*_eq_, since *T*_d_ = *R*_eq_·*C*·ln(*V*_DD_/*V*_T_) [12]; this is valid provided that *R*_eq_ is much higher than the parasitic output resistance of the I/O pins involved in the measurement. For a given *T*_d_ and assuming that *C*, *V*_DD_ and *V*_T_ are known, *R*_eq_ can be estimated as

(1) $$R_{\mathrm{eq}}=\frac{T_{d}}{C\mathrm{In}(V_{\mathrm{DD}}/V_{T})}$$

Unlike the circuits proposed in [12,13], the circuit in Figure 2a does not require any reference resistor because the information to be monitored is on the change, not in the absolute value.

### 3.2. Read-out Circuit with a Smart Wake-up

**Figure 3:**
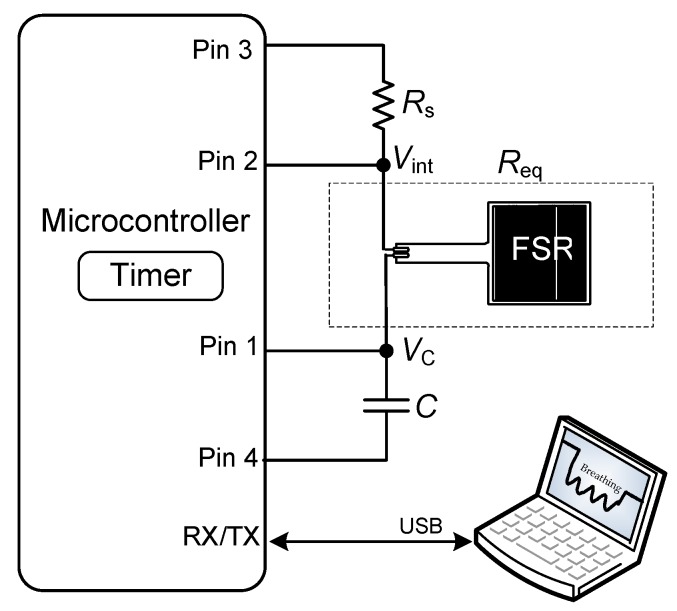
DIC applied to measure the FSR with a smart wake-up.

The main limitation of the circuit shown in Figure 2a is that the presence of a subject/object over the chair is detected by polling, thus involving unnecessary energy consumption especially when the seat is vacant. This can be improved using the DIC-based topology shown in Figure 3, which has a smart wake-up generated by the FSR itself when a sudden decrease of resistance occurs. In other words: the MCU is in a deep sleep mode by default, but when a sudden decrease of resistance happens, the MCU wakes up and measures the FSR in the same way indicated in Section 3.1. This kind of smart wake-up in MCU-based circuits was also proposed in [5,25], but employing a sensor for the wake-up and another for the measurement of interest. Here, the FSR is employed for both purposes.

The operating principle of the circuit shown in Figure 3 is the following. Initially, the MCU is in a deep sleep mode and the circuit is configured as a voltage divider using a series resistance (*R*_s_). The pins are configured as: Pin 3 provides a digital ‘1’, Pin 2 is set as external interruption with falling edge attention, Pin 1 provides a digital ‘0’, and Pin 4 is in HZ. Note that the current consumption of this voltage divider is very low since the FSR offers a very high resistance in zero-force conditions. When a subject/object is seated/placed over the FSR, its resistance suddenly decreases and, consequently, the output (*V*_int_) of the voltage divider also decreases, thus generating an external interruption attended by Pin 2. In such a case, the MCU wakes up and then measures the FSR as described in Section 3.1. In comparison with the circuit shown in Figure 2a, there are two differences: (1) Pin 3 is set in HZ and Pin 4 provides a digital ‘0’ during the charge-discharge process shown in Figure 2b, and (2) *R*_p_ is not required here since the sensor resistance is quite low when a subject/object is seated/placed over the chair and, hence, the discharging time is not expected to be so long. Therefore, in such a topology, we have *R*_eq_ = *R_x_* during the discharge process.

Once the MCU is awake, the measurement of *T*_d_ is repeated *n* times with a sampling frequency of *f*_s_ during an acquisition time of *T*_acq_ (= *n*/*f*_s_) that should be long enough to appropriately monitor the respiratory signal. The main energy consumption occurs when charging *C* to *V*_DD_ and when measuring *T*_d_ with a timer running at high frequency so as to reduce the effects of quantization. Accordingly, the energy required to carry out the complete measurement can be approximated as

(2) $$E\approx V_{\mathrm{DD}}C\left[V_{\mathrm{DD}}+I_{\mathrm{timer}}R_{\mathrm{eq}}\ln\left(\frac{V_{\mathrm{DD}}}{V_{T}}\right)\right]f_{s}T_{\mathrm{acq}}$$

where *I*_timer_ is the current consumption of the timer in charge of measuring *T*_d_. This is valid provided that the current consumption of the MCU while controlling both the charging stage and the sampling frequency is negligible. Note that these two tasks can easily be controlled by a timer running at low frequency (e.g., 32 kHz) [26] that generally involves a current consumption smaller than 1 µA. The consumption related to the data processing is not considered in Equation (2).

**Figure 4:**
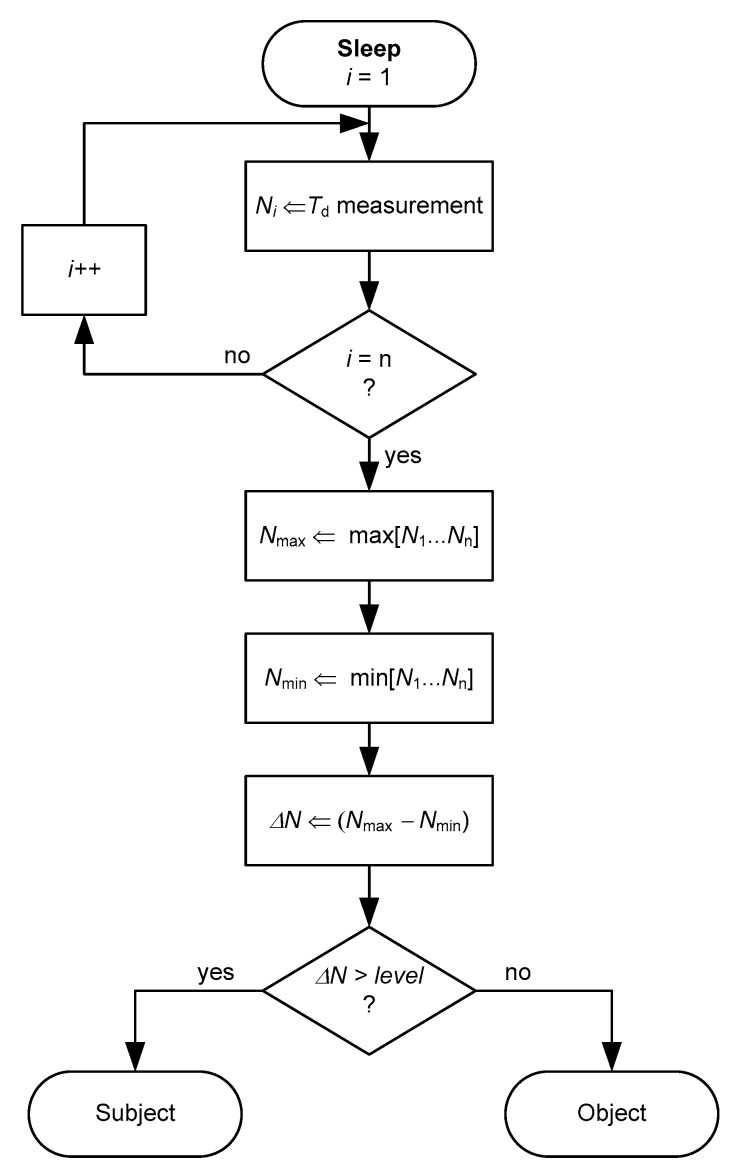
Flowchart of the proposed algorithm for the subject-object classification.

An algorithm to be executed by the MCU for the subject-object classification is proposed in Figure 4. When the MCU wakes up from the deep sleep mode, it measures *T*_d_ up to *n* times and the result is stored in a 16-bit variable *N*_i_. From the set of measurements, the maximum (*N*_max_) and minimum (*N*_min_) values are found; a low-pass filter processing of the data could be required before determine *N*_max_ and *N*_min_ so as to avoid the effects of aberrant measurements. Afterwards, the difference (Δ*N*) between *N*_max_ and *N*_min_ is calculated. If such a difference is higher than a given threshold level, the presence of a subject is confirmed. As explained later in Section 5.2, the circuit in Figure 3 is expected to operate at *f*_s_ = 2 Sa/s during *T*_acq_ = 10 s and, therefore, 20 samples of *T*_d_ (of 16 bits) will be stored. This involves 40 bytes of RAM, which are available in most MCUs.

## 4. Materials and Method

### 4.1. General

A commercial FSR (FSR 406 from Interlink Electronics) was arranged in the center of the seat of a conventional chair. This FSR has *R_x_*_,0_ > 10 MΩ, an active area of 4 × 4 cm^2^ and a rise time lower than 3 µs. The sensor was then connected to a DIC implemented by a low-cost, low-power MCU (MSP430F123 from Texas Instruments, Dallas, TX, USA) operating at 8 MHz and powered at *V*_DD_ = 3.3 V. This MCU has several Low-Power Modes (LPM) to control which on-chip circuitry, such as the central processing unit (CPU) and peripherals, is active. It also has 256 bytes of RAM.

An embedded 16-bit timer (running at 8 MHz and operating in LPM3) measured *T*_d_ as in Figure 2b. Several samples of *T*_d_ were taken at a sampling frequency of *f*_s_, which was controlled by another embedded timer. The frequency of the respiratory signal is expected to be very low (lower than 1 Hz) and, hence, *f*_s_ ≥ 2 Sa/s following the Nyquist criterion. Each sample of *T*_d_ was transmitted in real time to a personal computer and, then, converted to the corresponding value of *R*_eq_ applying Equation (1) through a control program implemented in LabVIEW^TM^.

**Figure 5:**
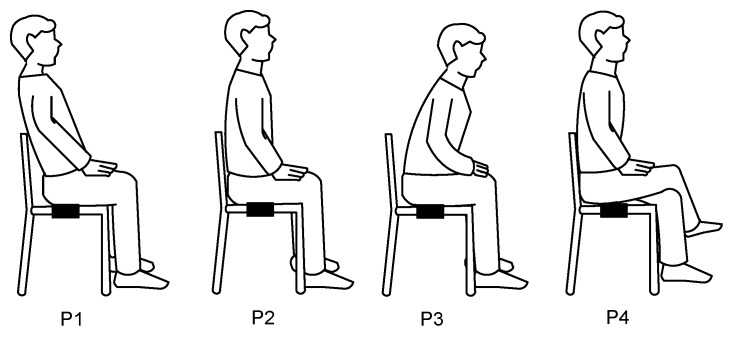
Different sitting postures of the subject to test the proposed measurement system.

The proposed measurement system was experimentally tested with three healthy subjects of different weight and age (S1: 45 kg/14 years; S2: 64 kg/39 years; S3: 91 kg/41 years), and also with three objects of different weight (O1: 5 kg; O2: 10 kg; O3: 30 kg). The volunteers were asked to position themselves over the chair and breathe freely, but to keep quiet during the measurement so as to avoid movement artifacts. Four different sitting postures of the subject were also tested, as shown in Figure 5. The idea of placing the FSR at the backrest was not considered because the signal detected would be insignificant in certain sitting postures, such as P3 in Figure 5.

The measurements of current consumption were carried out by a digital electrometer (Keithley 6514) following the procedures indicated in [26].

### 4.2. Basic Read-out Circuit

The circuit in Figure 2a was tested using *R*_p_ = 3570 Ω, and different values of *C* (470 nF, 1 µF and 2.2 µF) and *f*_s_ (2, 20 and 60 Sa/s). The values of *C* were high enough to have a good resolution in the measurement of *T*_d_ and, hence, of *R*_eq_ [27]. The selected value of *R*_p_ (together with the maximum value of *C*) generated *T*_d_ ≤ 8 ms, thus avoiding the overflow of the timer in zero-force conditions.

The function of Pin 1 and Pin 2 in Figure 2a was implemented by pins P1.1 and P1.2, respectively, which are general-purpose I/O digital pins. The former has a Schmitt-Trigger buffer with *V*_T_ = 1.2 V and is associated with a capture module that automatically captures the value of the timer when the external signal crosses *V*_T_.

### 4.3. Read-out Circuit with a Smart-Wake-up

In the circuit shown in Figure 3, the MCU operated by default in LPM4, where the CPU and all clocks are disabled and only the external interruption generated by Pin 2 is enabled; the wake-up time from LPM4 is 6 µs, which is fast enough for the application considered herein. Moreover, the circuit employed *R*_s_ = 1 MΩ, which is more than ten times smaller than the FSR resistance in zero-force conditions, thus generating a digital ’1’ at the input of Pin 2 when the seat was vacant. The circuit was optimized in terms of energy consumption using the minimum values of *C* (470 nF) and *f*_s_ (2 Sa/s) from those indicated in Section 4.2, as suggested by Equation (2).

The function of pins 1, 2, 3 and 4 in Figure 3 was implemented by pins P1.1, P1.2, P1.3 and P1.4, respectively. In addition to the features indicated in the second paragraph of Section 4.2, P1.2 was configured as an external interruption to automatically wake the MCU from LPM4 when the FSR underwent a sudden change.

## 5. Experimental Results and Discussion

### 5.1. Basic Read-out Circuit

**Figure 6:**
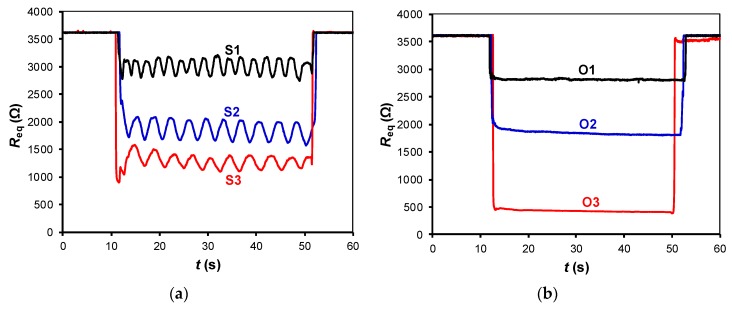
Experimental resistance variations monitored by the DIC in Figure 2a when over the chair we had: (**a**) subjects of different weight and age in the sitting posture P2; (**b**) objects of different weight. The test was carried out at *f*_s_ = 60 Sa/s with *C* = 2.2 µF.

The experimental results of the circuit in Figure 2a for different subjects are shown in Figure 6a. When a subject sat down (at *t* ≈ 10 s), a large-signal variation (between 600 and 2400 Ω) was observed, and this depended on the subject’s weight. For the heaviest subject (S3), the resistance dropped to an average value of about 1250 Ω. While seated, small-signal resistance variations of around ±125 Ω with a respiratory rate between 12 (for S3) and 24 (for S1) breaths per minute, which correspond to a frequency between 0.2 and 0.4 Hz, were monitored. This well-defined respiratory signal clearly confirms the presence of a subject over the chair without incorporating any other sensor.

The results when placing different objects over the chair are represented in Figure 6b, which only shows a large-signal variation due to the object’s weight but not the small-signal variations. It is worth mentioning that in Figure 6b the large-signal variation is similar and even higher than that in Figure 6a although the weight is lower. This is because the objects under test had small dimensions and, consequently, their weight caused a “point” force on the FSR that was higher than the corresponding component of distributed force generated by the subject.

**Figure 7:**
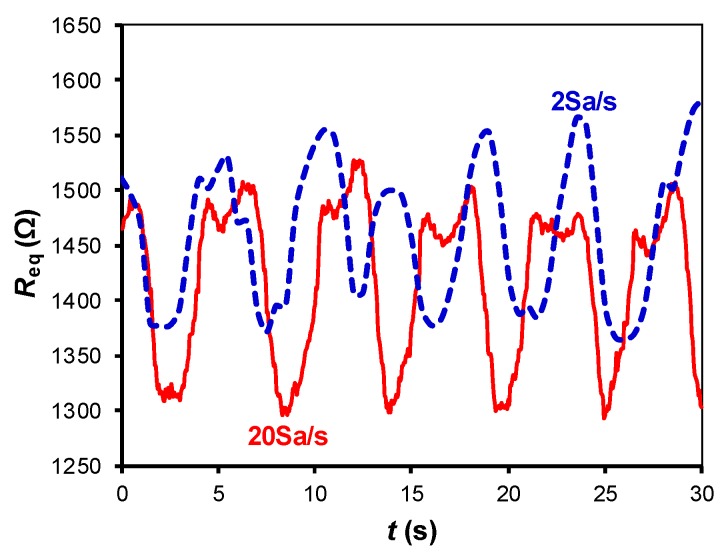
Small-signal resistance variations monitored by the DIC in Figure 2a for different sampling frequencies. The test was carried with subject S3 in the sitting posture P2, with *C* = 1 µF.

**Figure 8:**
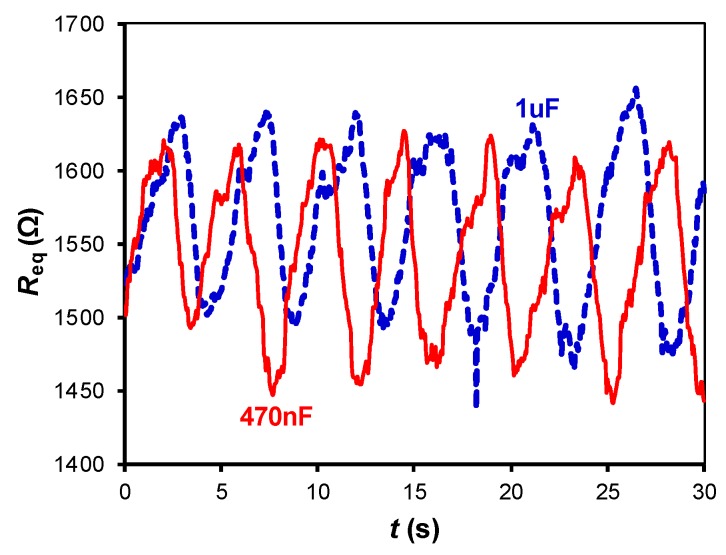
Small-signal resistance variations monitored by the DIC in Figure 2a for different values of the capacitor. The test was carried with subject S2 in the sitting posture P2, with *f*_s_ = 20 Sa/s.

**Figure 9:**
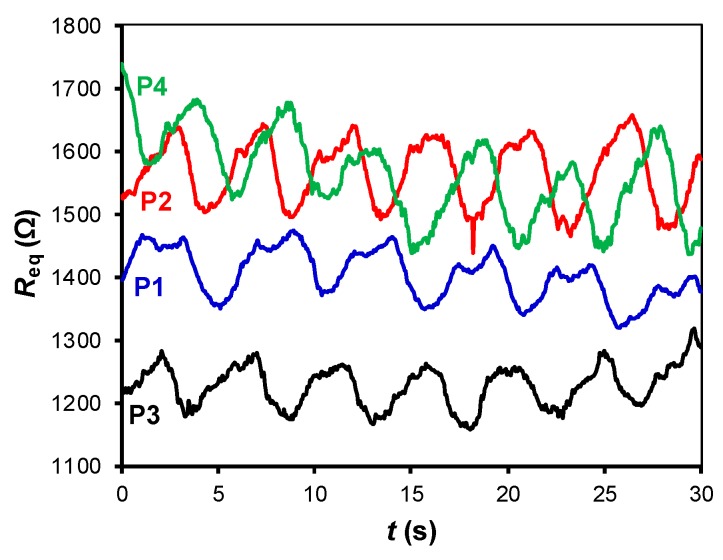
Small-signal resistance variations monitored by the DIC in Figure 2a for different sitting postures (see Figure 5). The test was carried with subject S2 at *f*_s_ = 20 Sa/s and with *C* = 1 µF.

Figure 7, Figure 8 and Figure 9 show the small-signal resistance variations monitored for different values of *f*_s_, *C* and sitting posture, respectively. According to Figure 7, a sampling frequency of 2 Sa/s seems to be enough to recover the frequency of the respiratory signal, with the corresponding benefits in terms of energy consumption. From Figure 8, a low-value capacitor (470 nF) seems valid to detect the resistance variations involved in the measurement, thus reducing the energy consumption of the circuit even more. According to Figure 9, the amplitude of the small-signal resistance variation was quite similar for the four sitting postures under test. Posture P3 generated the highest large-signal variation at the instant at which the seat was occupied, whereas posture P4 provided a more unstable signal probably because of the crossed leg.

### 5.2. Read-out Circuit with a Smart Wake-up

**Figure 10:**
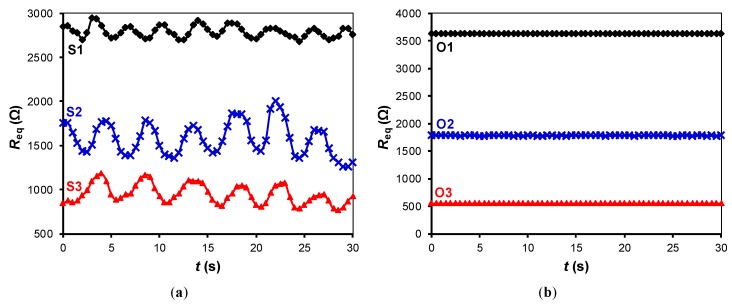
Experimental resistance variations monitored by the DIC in Figure 3 when over the chair we had: (**a**) subjects of different weight and age in the sitting posture P2; (**b**) objects of different weight. The test was carried out at *f*_s_ = 2 Sa/s with *C* = 470 nF.

**Figure 11:**
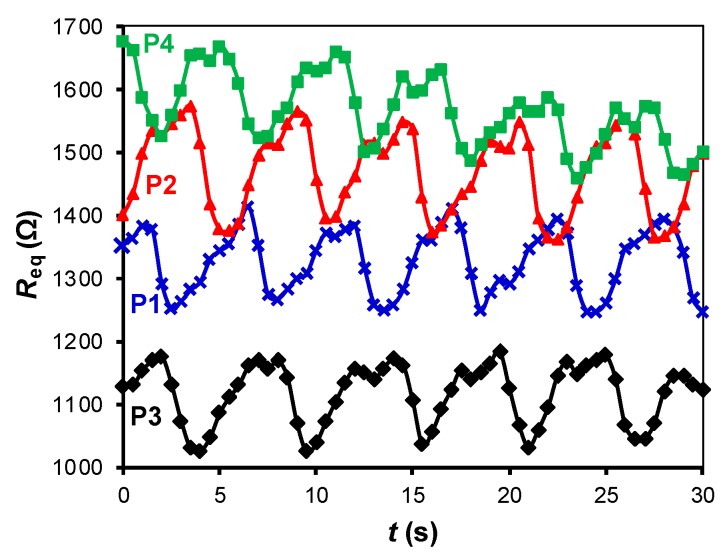
Small-signal resistance variations monitored by the DIC in Figure 3 for different sitting postures (see Figure 5). The test was carried with subject S2 at *f*_s_ = 2 Sa/s and with *C* = 470 nF.

The experimental results of the circuit in Figure 3 for different subjects and objects are shown in Figure 10a,b, respectively, assuming that *t* = 0 is the instant at which the subject/object is seated/placed over the chair, thus waking up the MCU. Although the values of *C* and *f*_s_ were low so as to reduce the energy consumption, the circuit was able to recover the frequency of the respiratory signal and to detect the resistance variations caused by respiration, as clearly shown in Figure 10a. If an object was placed over the chair instead of a subject, the resulting *R*_eq_ was almost constant over time after the wake-up, as shown in Figure 10b. Accordingly, the subject-object classification is also feasible with the circuit in Figure 3. An appropriate threshold level (see Figure 4) for the classification could be, for instance, 50 Ω, which corresponds to Δ*N* = 188 counts under the operating conditions indicated before. From Figure 10a, the acquisition time should be around 10 s in order to have enough samples (even for very low respiratory rates) that enable the detection of the respiratory signal. The effects of the sitting posture when using the circuit in Figure 3 are represented in Figure 11, which shows a performance quite similar to that represented before in Figure 9.

The current consumption of the circuit shown in Figure 3 in LPM4 (i.e., when waiting for an interruption generated by a subject/object) was 800 nA, including the current of the voltage divider formed by *R*_s_ and FSR. On the other hand, the current consumption of the embedded timer (at 8 MHz and in LPM3) while measuring *T*_d_ was 500 µA. According to Equation (2) and assuming *V*_DD_ = 3.3 V, *V*_T_ = 1.2 V, *C* = 470 nF, *I*_timer_ = 500 µA, *R*_eq_ = 1600 Ω, *f*_s_ = 2 Sa/s, and *T*_acq_ = 10 s, the energy required is 125 µJ. Therefore, in case of using a lithium battery of 3.6 V − 1 Ah, the circuit in Figure 3 would have autonomy to check more than 100 million times if the interruption was generated by a subject or an object. Taking into account that the circuit in Figure 3 was clearly able to detect the resistance variations caused by respiration, the discharging-time measurement could also be carried out at a lower operating frequency (e.g., 1 MHz instead of 8 MHz), thus reducing even more the energy required since *I*_timer_ would be smaller. However, the previous estimation of the autonomy would be shorter if the system had a transceiver circuit whose energy consumption can be quite significant.

## 6. Conclusions

This work has proved that detecting and confirming the presence of a subject in a chair is feasible using a single FSR directly connected to a general-purpose MCU. The proposed system first detects the subject by monitoring his/her weight and then confirms his/her presence by monitoring the respiration. The proposed MCU-based circuit has also been improved in terms of energy consumption by incorporating a smart wake-up generated by the FSR itself. In such a way, the MCU is by default in a deep sleep mode. We believe the proposed system is suitable for applications related to autonomous sensors where it is important to detect and confirm the presence of people sitting in chairs, such as intelligent airbag deployment systems and aircraft boarding systems.
